# Research progress on HER2-specific chimeric antigen receptor T cells for immunotherapy of solid tumors

**DOI:** 10.3389/fimmu.2025.1514994

**Published:** 2025-05-21

**Authors:** Liaoliao Zhu, Jingyi Liu, Junqiang Li, Nan Wang, Yan Zhao, Haichuan Su

**Affiliations:** State Key Laboratory of Holistic Integrative Management of Gastrointestinal Cancers, Department of Oncology, Tangdu Hospital, Air Force Medical University, Xi’an, Shannxi, China

**Keywords:** CAR-T, HER2, solid tumors, CARs structure, optimization strategies, clinical application

## Abstract

Human epidermal growth factor receptor 2 (HER2) is highly expressed in various solid tumors, and its abnormal activation is closely associated with poor tumor prognosis, establishing it as a prominent target in contemporary research. The successful clinical treatment of multiple HER2-positive tumors with HER2 antibodies has prompted researchers to design chimeric antigen receptor T (CAR-T) cells targeting HER2 for solid tumor immunotherapy. To date, the development of CAR structures has progressed to the fifth generation, with most HER2-CAR-T cell structures being modified based on the second-generation CAR architecture. This review will delineate the structure and cytotoxic mechanism of HER2-CAR-T cells, elucidate the difficulties and optimization strategies for HER2-CAR-T cell therapy, and summarize recent clinical applications and advancements.

## 1 Introduction

Since 1987 (1), when it was discovered that human epidermal growth factor receptor 2 (HER2) amplification or overexpression in patients with breast cancer was correlated with shorter survival periods, identifying it as a key oncogenic driver, research on the HER2 gene has been relentless. HER2, a tyrosine kinase receptor located on the cell surface in its monomeric form (2), has been strongly implicated in poor prognosis across various solid tumors, including those of the gastric, endometrial, bladder, lung, colon, prostate, and head and neck regions (3). HER2 amplification or overexpression is observed in approximately 15-30% of breast cancers and 10-30% of gastric/gastroesophageal cancers, serving as both a prognostic and predictive biomarker (4). HER2 plays a critical role in processes such as cell proliferation, differentiation, and angiogenesis. Its high expression can accelerate cell division, disrupt the balance between proliferation and differentiation, and ultimately lead to cancer cell transformation (5). Moreover, high HER2 expression has been shown to increase migration and invasion rates, interfere with adhesion molecule synthesis, and promote tumor invasion, metastasis, and recurrence (6, 7). In 1998, trastuzumab, the first anti-HER2 monoclonal antibody, received Food and Drug Administration (FDA) approval, significantly improving the prognosis of patients with HER2-positive metastatic breast cancer (8). Targeted HER2 therapy has also substantially extended the survival of patients with advanced HER2-positive gastric cancer (9, 10). However, many patients with HER2-positive solid tumors develop resistance to HER2 monoclonal antibodies (e.g., trastuzumab), and the associated cardiotoxicity and other side effects limit their clinical application (11, 12). Consequently, researchers have explored alternative HER2-targeted therapies, with CAR-T cell therapy gaining favor due to its notable clinical efficacy in treating hematological cancers.

Chimeric antigen receptor T (CAR-T) cell therapy involves using genetic modification techniques to transfer genetic material encoding specific antigen recognition domains and T cell activation signals into T cells. These modified T cells can directly bind to specific antigens on tumor cell surfaces and become activated, killing tumor cells by releasing perforin, granzyme B, and other cytokines, and recruiting endogenous immune cells for further tumor cell destruction, thereby achieving therapeutic goals (13, 14). This therapy also generates immune memory T cells, providing long-term anti-tumor effects (13). In hematological malignancies, the most commonly targeted proteins include CD19, BCMA, and CD22, whereas in solid tumors, tumor-associated antigens (TAA), HER2, and mesothelin (MSLN) are frequently targeted (15). HER2-CAR-T therapy has shown breakthrough progress in various solid tumors such as glioblastoma (16), breast cancer (17), colorectal cancer (18), and gastric cancer (19).

This review will first discuss the various designs of HER2-specific CAR structures in previous studies and their therapeutic effects on solid tumors. Secondly, it will elucidate the mechanisms by which HER2-CAR T cells exert their effects, the current challenges they face, and optimization strategies. Finally, it will summarize the clinical research findings on HER2-CAR T cells in solid tumors over the past decade.

## 2 HER2-specifc CAR structure and optimization

**Figure 1:**
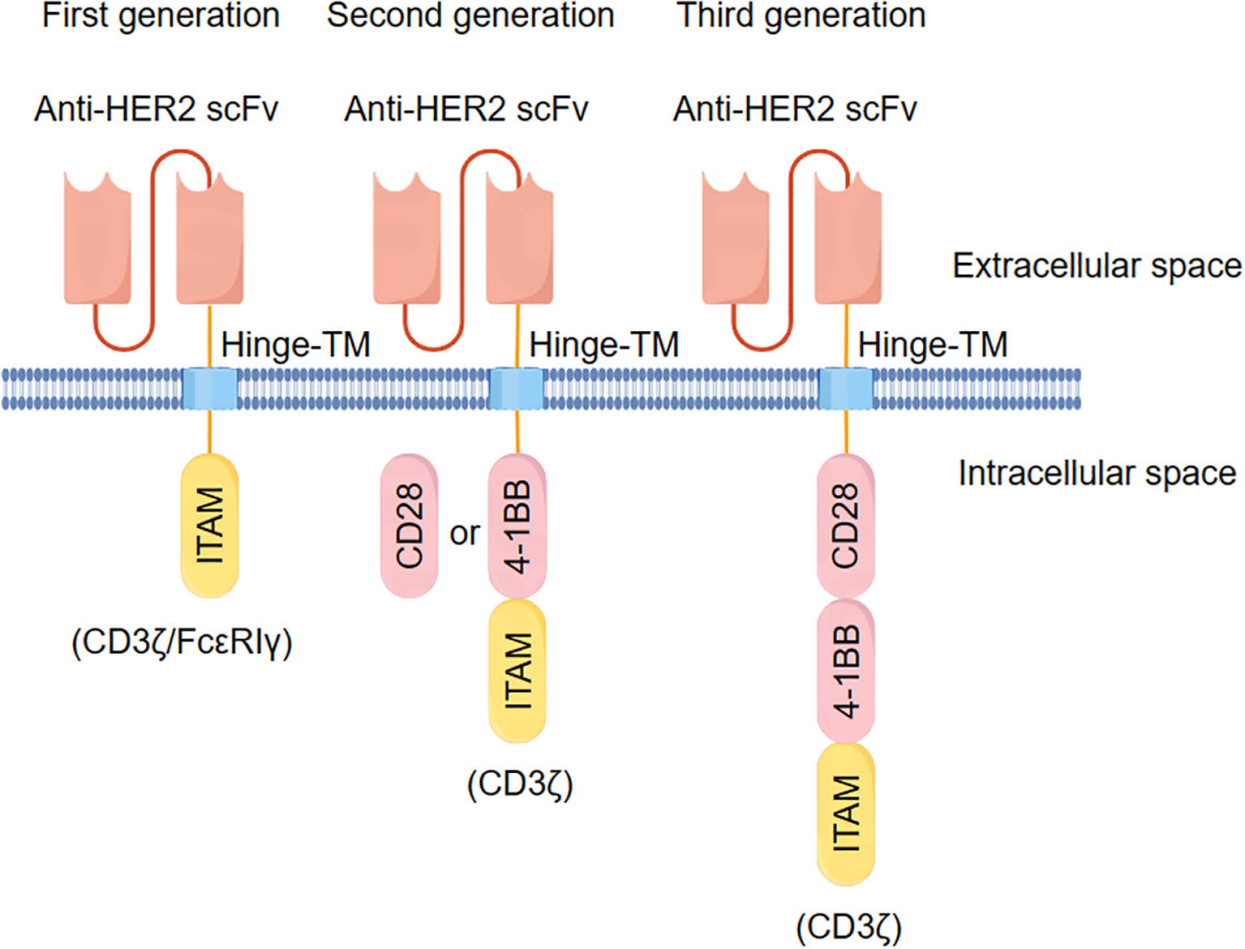
The current universal HER2 specific CAR molecular structure.

According to the intracellular domain of CAR, research on CAR design has advanced to the fifth generation (20). The HER2-specific CAR structure has been studied from the first generation to the third generation (**Figure 1**). The signal domain of the first-generation CAR design consisted of a single signal molecule, connected by a single-chain antibody through a transmembrane region to the immune receptor tyrosine-based activation motif (ITAM) in the intracellular signal transduction region (21). In 1993, researchers first constructed the first-generation HER2-specific CAR, which incorporated the antigen-binding domain of anti-HER2 antibodies and the TCR/CD3 complex ζ signal transduction subunits or Ig-Fc receptor complexes γ signal transduction subunit. Subsequently, it was discovered that engineered CAR-T cells specifically recognize HER2 and secrete cytokine interleukin-2 (IL-2) (22). However, the expression of co-stimulatory molecules on the surface of tumor cells is often weakened or absent, causing CAR-modified T cells to lack the support of co-stimulatory second signals. After T-cell activation, they quickly lose their function due to signal limitations. This defect leads to CAR-T cells being unable to expand effectively, causing premature cell aging. Although these cells have a killing effect, they are difficult to sustain, which limits the anti-tumor efficacy of first-generation CAR-T cells in patients (23).

Based on this, the second-generation CAR introduces a co-stimulatory domain such as CD28 (24) or CD137 (4-1BB) (25), significantly enhancing CAR-T immune activity activation and persistence in clinical trials. Researchers constructed a humanized HER2-CAR containing the chA21 single-chain variable fragment (scFv) region of an antigen-specific monoclonal antibody and T-cell intracellular signaling chains composed of CD28 and CD3ζ. This structure can not only recognize and kill HER2-positive breast and ovarian cancer cells but also lead to the disappearance of breast cancer cells *in vivo (* 26). A study comparing the functional activity of HER2-CAR constructs containing CD28 or 4-1BB intracellular co-stimulatory signal domains found that HER2-CARs with 4-1BB co-stimulatory domains exhibited improved tumor targeting, reduced T cell depletion, and enhanced proliferation ability, making them suitable for treating multifocal brain metastases (27). Researchers discovered that second-generation HER2-specific CAR-T cells exhibited strong cytotoxicity and cytokine-secreting ability against colorectal cancer (CRC) cells *in vitro*. These cells also displayed greater aggressiveness in HER2-positive CRC in patient-derived tumor xenograft (PDX) models and showed potent immunotherapeutic capacity for CRC in metastatic xenograft mouse models (28).

**Figure 2:**
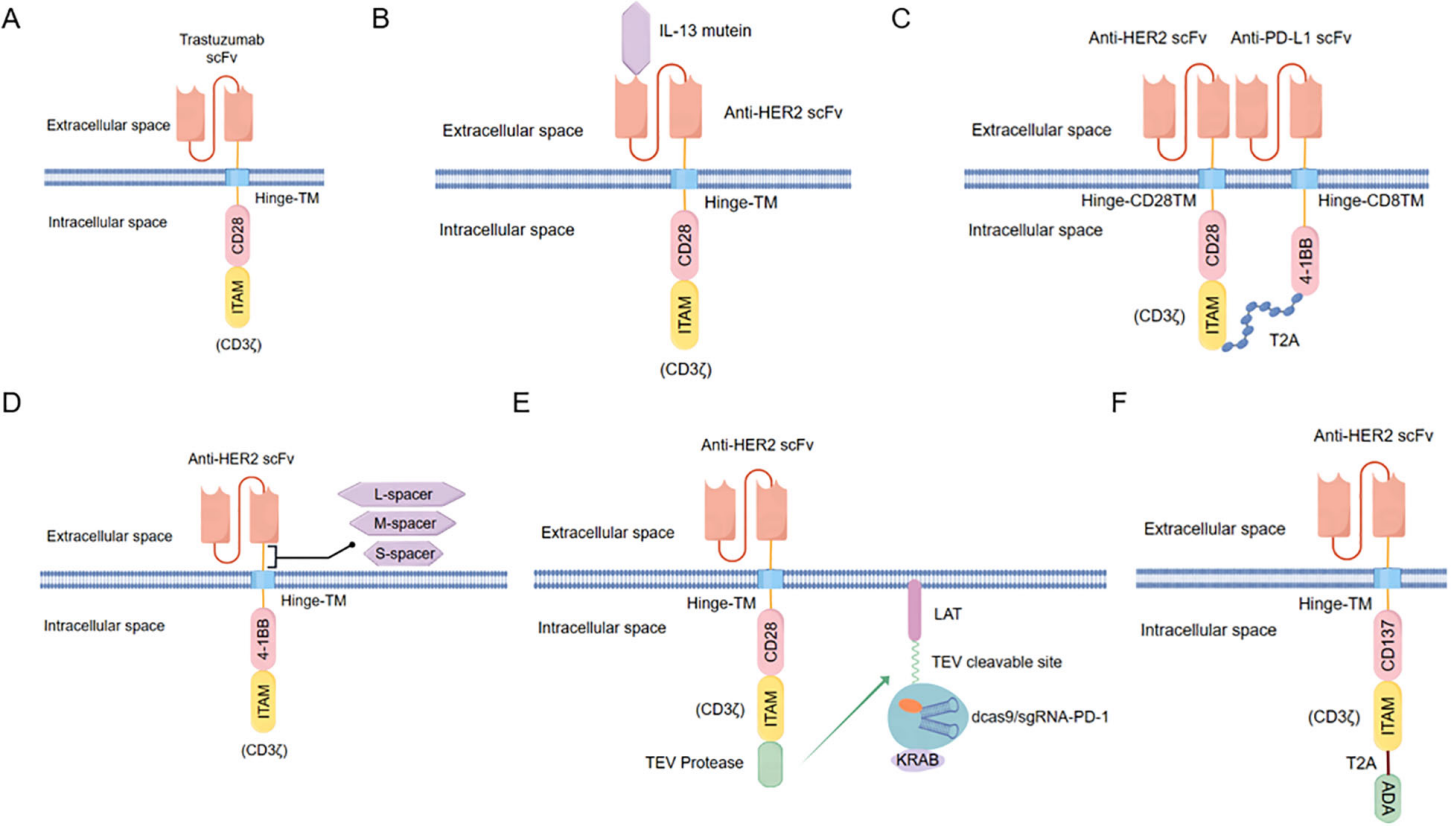
Design of optimized molecular structures for HER2-specific CAR molecules reported. **(A)** Screening of scFv fragments using the monoclonal antibody trastuzumab against HER2, which binds to second-generation CAR. **(B)** Bispecific CAR containing anti-HER2 scFv and an IL13Rα2-binding IL-13 mutein. **(C)** Dual-targeting CAR co-expresses anti-PD-L1 and anti-HER2 scFv, with the intracellular structure connected by peptide T2A. **(D)** Three types of HER2-specific CARs designed based on the length of the spacer sequence connecting the scFv and transmembrane region (TM). **(E)** HER2-specific CAR with an additional construct containing linker for activation of T cells (LAT), a tobacco etch virus (TEV) cleavable sequence (TCS), nuclease-deactivated spCas9 (dCas9), and Kruppel associated box (KRAB). Upon antigen presence, TEV protease cleaves TCS, allowing dCas9-KRAB to target the PD-1 transcription start site. **(F)** HER2-specific CAR intracellular domain CD3ζ connects ADA through peptide T2A.

In addition, research on HER2-CAR structures has led to the development of CARs with an scFv binding domain derived from trastuzumab and cytoplasmic regions of human CD28 and CD3ζ signaling domains (**Figure 2A**). HER2-CAR-T cells have demonstrated the ability to penetrate the tumor matrix, significantly improving long-term survival rates in mice (29). Even small quantities of HER2-CAR-T cells can elicit a robust immune response, ultimately leading to tumor remission (30). Researchers have also engineered a bispecific CAR combining a HER2-binding scFv and an IL13Rα2-binding IL-13 mutein (**Figure 2B**), which effectively reduces antigen escape and enhances T cell anti-tumor activity (31). Moreover, a novel dual-targeting CAR-T cell was designed to co-express a PD-L1-targeting chimeric switch receptor (PD-L1.BB CSR) and a HER2.28ζ CAR (**Figure 2C**). These HER2.28ζ/PD-L1.BB CAR-T cells demonstrated rapid and durable eradication of pleural and peritoneal metastatic tumors in xenograft models, leading to the initiation of a phase I clinical trial for patients with pleural or peritoneal metastasis (NCT04684459) (32). In gastric cancer research, a novel CAR-T targeting HER2, containing CD137 and CD3ζ, showed significantly enhanced tumor inhibition, long-term survival, and targeted homing capabilities (33). Professor Nicholas A. Vitanza’s group developed HER2-specific CAR-T cells with medium-length CAR spacer sequences (**Figure 2D**). The spacer sequence, a non-antigen-binding extracellular domain, connects the scFV and transmembrane regions (TM). Varying the spacer length influenced CAR activity, with medium-length spacers demonstrating superior efficacy in HER2-positive neural tumor cells in glioma mouse models (34). Additionally, HER2-CAR-T cells combined with CRISPR interference have been constructed. The anti-HER2 scFV extracellular segment connects with CD28 and CD3ζ co-stimulatory domains linked to the tobacco etch virus (TEV) protease and a single guide RNA (sgRNA) targeting the PD-1 transcription start site (TSS). A second construct includes a linker for activation of T cells (LAT) fused to nuclease-deactivated spCas9 (dCas9)-Kruppel-associated box (KRAB) *via* a TEV-cleavable sequence (TCS). Upon antigen encounter, the LAT-dCas9-KRAB (LdCK) complex is cleaved by TEV, allowing targeting of dCas9-KRAB to the PD-1 gene TSS (**Figure 2E**). This HER2-CAR-T cell construct has demonstrated superior cancer growth inhibition compared to traditional CAR-T cells (35). A research group also screened for the metabolic regulator ADA, overexpressing it in the same lentiviral vector as the HER2-CAR under a T2A (self-cleaving peptide) element (**Figure 2F**). ADA overexpression improved tumor infiltration and clearance by HER2-specific CAR-T cells in an *in vivo* colorectal cancer model (36). Currently, the CAR-T products available on the market are all second-generation CAR-T products.

The third-generation CAR-T technology incorporates an additional co-stimulatory molecule on the basis of the second generation, enhancing both proliferation and lethality (37). It primarily includes two co-stimulatory domains: one being CD28 or 4-1BB, and the other OX40, CD28, or 4-1BB (38). Although third-generation CARs have demonstrated stronger and more persistent activity in some preclinical trials (39) and clinical data has confirmed their safety and efficacy for human use (40), there are reports suggesting that third-generation CARs may lower the T cell stimulation threshold, leading to signal leakage and potentially excessive cytokine release. In comparison, second-generation CARs can activate other CD3 signal transduction sources, potentially providing stronger signal transduction and higher anti-tumor effects (41). A recent study constructed third-generation CAR-T cells targeting the HER2 antigen in glioblastoma, demonstrating effective anti-tumor activity both *in vitro* and *in vivo (* 42).

The fourth-generation CAR-T technology incorporates suicide genes and modifies immune factors to prevent cytokine storm side effects and finely regulate the immune response (43). Some studies have added cytokine or chemokine receptor structures to increase T-cell infiltration in tumor tissue, thereby enhancing the killing effect on solid tumors (44). The fifth-generation CAR-T technology aims to overcome individual limitations, offering universal applicability across different patients with large-scale production and treatment potential (45, 46). However, this general-purpose CAR-T technology faces high technical barriers and stringent safety requirements (47). Currently, there are no reports on the construction of fourth and fifth-generation CARs targeting HER2.

Previous studies on CAR structure optimization have indicated that using a CD3ζ chain with only one ITAM domain, rather than the entire length, can achieve stronger tumor inhibitory activity (48). Researchers have also discovered that CAR constructs containing CD3δ, CD3ϵ, or CD3γ perform better *in vivo* compared to traditional CD3ζ CAR-T cells (49). While many studies have focused on optimizing CAR-T cell structure and enhancing their anti-tumor effects, the true efficacy still needs to be verified in clinical trials.

## 3 Difficulties and optimization strategies of HER2-CAR-T therapy for solid tumors

**Figure 3:**
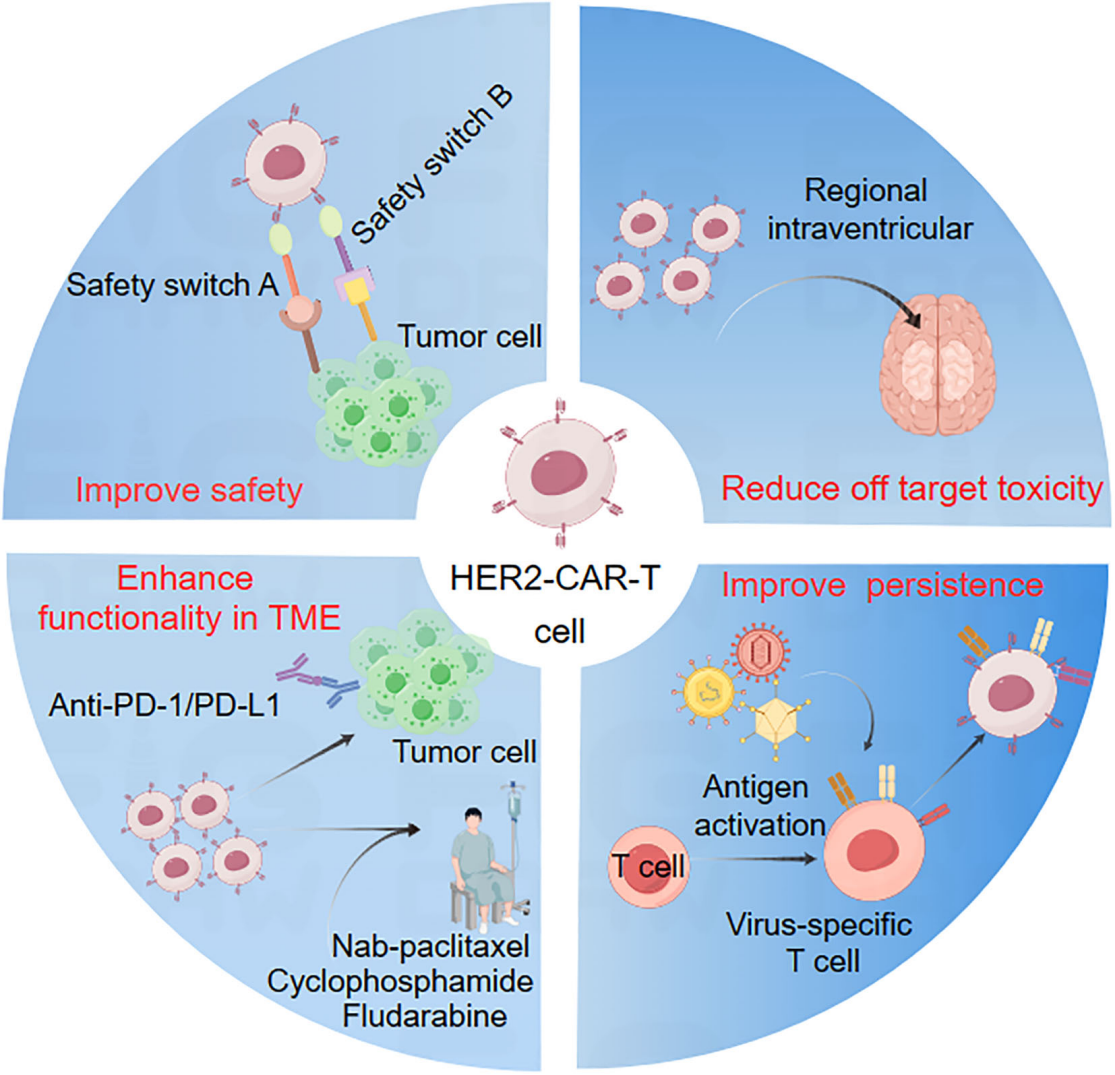
Partial optimization strategies for HER2-CAR-T cell therapy. Firstly, the various delivery methods of HER2-CAR-T cells result in differential effectiveness. Regional intraventricular delivery therapy, for instance, may effectively address the challenge of drugs crossing the blood-brain barrier, particularly in the treatment of brain tumors or solid tumor brain metastases (top right). Secondly, this is a bifunctional/bispecific safety switch based on small molecules, consisting of two key modules: one with the ability to recognize and bind specifically to TAA, and the other with the ability to bind to the antigen recognition domain reprogrammed by CAR. This safety switch acts as a quasi immune synapse between CAR-T cells and tumor cells in a time-dependent or dose-dependent manner, transforming CAR-T cells into an “on” state. Only after administering a small molecule safety switch can a quasi immune synapse be formed, triggering CAR-T cells to eliminate target cancer cells (top left). Thirdly, combining CAR-T therapy with chemotherapy is crucial in controlling disease progression and reducing tumor burden. Chemotherapy, typically administered 3–5 days before CAR-T cell infusion, creates an immune microenvironment favorable for CAR-T cell engraftment, expansion, and survival. Furthermore, the integration of CAR-T cells with immune checkpoint inhibitors (ICIs) can diminish the immunosuppressive effects of the tumor microenvironment, thereby improving CAR-T cell efficacy and prolonging survival (lower left). Lastly, virus-specific T cells (VSTs) utilize viral surface protein antigens to stimulate T cells *in vitro*, enabling the acquisition and analysis of antigen-specific T cells. VSTs not only recognize and eliminate infected cells but also activate other components of the immune system to coordinate a comprehensive response. CAR-T cells derived from VSTs combine CAR’s anti-tumor activity while mitigating the risk of graft-versus-host disease (GvHD) (lower right).

Although HER2-CAR-T with optimized structure has achieved preliminary efficacy in the treatment of solid tumors, its efficacy is inferior to that of hematological tumors. This is mainly related to the characteristics of solid tumors. Compared with hematological tumors, the treatment of solid tumors needs to solve more difficulties. Researchers have designed various optimization strategies to address these obstacles (**Figure 3**).

### 3.1 On-target, off-tumor toxicity

Unlike blood tumors, which often have a single and specific target, tumor specific antigens (TSA) are rare in solid tumors. Currently, the antigens found to be highly expressed in tumors are mostly tumor associated antigens (TAA), which are also expressed in normal tissues, this leads to CAR-T cells damaging normal tissue cells when specifically targeting tumor cells. This brings high off target risks, and safety becomes a key issue. Although HER2 is highly expressed in various solid tumors, its heterogeneous expression within tumors can lead to poor therapeutic effects of HER2-CAR-T cells. For example, a patient with advanced colon cancer accompanied by liver and lung metastasis died after receiving HER2-CAR-T therapy for 5 days. The attack of CAR-T on lung epithelial cells expressing low levels of HER2 is considered the main cause of death, resulting in a high level of cytokines in the blood (50). In addition, single target HER2-CAR-T is difficult to eradicate all solid tumor cells in the same tumor lesion (51). So, what are the current strategies for alleviating off-tumor targeted toxicity?

Researchers have summarized and introduced in detail strategies to better limit the off-tumor cytotoxicity of CAR-T cells (52). Among them, some researchers have increased the specificity and sensitivity of CAR-T antigen recognition by distinguishing the difference in TAA density between tumor tissue and normal tissue. Wendell A. Lim’s team’s research shows that a highly sensitive antigen density sensitive CAR-T was constructed using a synNotch (synthetic Notch) receptor system, in which a low affinity synNotch receptor targeting HER2 controls the expression of a high affinity CAR targeting HER2. When the synNotch receptor is fully activated by high antigen density HER2, it induces the expression of high affinity CARs, which then initiate CAR-T specific killing of tumor cells. Through *in vitro* and *in vivo* studies, it has been confirmed that CAR-T cells expressing this system have significant differences in killing non-tumor cells expressing normal amounts of HER2 and tumor cells expressing 100 fold HER2 (53). The synNotch technology can increase the specificity and sensitivity of CAR-T antigen recognition by distinguishing the difference in TAA density between tumor tissue and normal tissue. At present, researchers have proposed a method for achieving expression level dependent tumor targeting through engineered antibody affinity (Avidity), which enhances the selectivity of nanobodies for high expression level targets through multivalent binding without binding to low expression targets in normal tissues, to some extent solving the problem of “on target, off tumor” (54). In addition, local delivery of CAR-T cells can also reduce off target tumor toxicity. Priceman et al. developed second-generation HER2-CAR-T cells and investigated their administration *via* intravenous, local intratumoral, and regional intraventricular routes in preclinical settings using human xenograft models of HER2-positive breast cancer with brain metastases. They observed a pronounced *in vivo* antitumor effect following local intracranial administration of HER2-CAR-T cells in an orthotopic model of brain metastasis. This intraventricular delivery approach could potentially overcome the challenge of the blood-brain barrier in treating brain tumor metastases (27).

For the high heterogeneity of solid tumors, targeting two or more tumor antigens simultaneously is an effective solution. For example, scFv sequences of HER2 antibodies with different affinities can be screened and combined with scFv structures of other antigen targets to construct tandem CAR (55), dual CAR (56), loop CAR (57), synNotch CAR, or trivalent CAR (58). In addition, HER2-CAR-T can be combined with bispecific antibodies, such as bispecific antibodies Bi specific T-cell adaptor (BiTE), which can simultaneously bind to CD3 molecules on the surface of T cells and HER2 antigen molecules on the surface of tumor cells, thereby inducing T cells to kill tumor cells. Choi et al. designed an anti-EGFR vIII CAR-T with autocrine anti-EGFR BiTE and demonstrated its ability to eradicate heterogeneous tumor cells in a glioblastoma mouse model (59).

### 3.2 Inhibition of immune microenvironment in solid tumors

Solid tumors have an extremely complex microenvironment that inhibits the infiltration, activity, and function of CAR-T cells to varying degrees. The physical barriers generated by tumor stromal cells and abnormal tumor vasculature limit the ability of CAR-T cells to reach the target site, leading to possible functional failure or reduced numbers before reaching the core area of the tumor (60). In addition, there are a large number of inhibitory cytokines (TGF-β, VEGF, IL-4, IL-10, etc.) and inhibitory cells (Treg, MDSC, etc.) in the tumor microenvironment, which can inhibit the activity and function of CAR-T cells (61). Furthermore, abnormal tumor metabolic microenvironments, such as metabolite accumulation, hypoxia, low pH, oxidative stress, etc., can affect the infiltration and activity of CAR-T cells (62).

Tumor anti-angiogenesis therapy and matrix degradation therapy can directly promote CAR-T cell infiltration. There are studies utilizing synthetic biology techniques to express synNotch receptors in HER2 targeted CAR-T cells to recognize tumor extracellular matrix and regulate the expression of matrix degrading enzymes. This new type of CAR-T cell can achieve precise regulation of the spatiotemporal specificity of matrix degrading enzyme expression, that is, only when CAR-T cells reach the tumor site, can they induce the expression and secretion of enzyme proteins, leading to the degradation of extracellular matrix components in tumor cells. CAR-T cells expressing synNotch receptors exhibit excellent matrix degradation and infiltration abilities, enhancing the infiltration and proliferation of CAR-T cells in mouse models, and clinical tumor patient derived organoids, while continuously inhibiting tumor growth (63). In addition, various traditional therapies, such as chemotherapy (64, 65), radiotherapy (66), and hyperthermia (67), can promote the infiltration of CAR-T cells due to their reshaping effect on tumor microenvironment.

The suppression or clearance of immune suppressive cells is one of the effective ways to maintain the function of tumor infiltrating CAR-T cells. Researchers combined oncolytic adenovirus (OAd) expressing TNF-α and IL-2 with CAR-T and found that it can promote M1 polarization of tumor associated macrophages and increase DC cell maturation, thereby significantly enhancing CAR-T infiltration and anti-tumor effects (68). Combining HER2-CAR-T cells with oncolytic virus (OV) delivery to the tumor microenvironment represents another optimization strategy (69). One research group successfully loaded HER2-CAR-T cells with OV, allowing OV delivery to tumor cells without impairing CAR-T function *in vitro*, thereby enhancing the tumoricidal activity of the combined treatment (70). However, the *in vivo* application of this method may be restricted to immunothermal tumors, as OV cannot enhance infiltration if HER2-CAR-T cells do not first infiltrate the tumor to deposit OV. Another group engineered an OV armed with a bispecific tumor-targeted T cell engager molecule CD44, IL-12, and a PD-L1-blocking antibody to boost HER2-CAR-T cell function. This combination significantly improved tumor control and survival (71).

In addition, CAR-T combined with immune checkpoint inhibitors (ICIs) has been widely proven to effectively enhance the control of solid tumors by CAR-T. One study evaluated the efficacy of third-generation anti-HER2 CAR-T cells alone and in combination with anti-PD-1 antibodies against HER2-expressing breast tumor cells *in vitro* and in immune mouse models. The results demonstrated that anti-PD-1 antibodies can significantly enhance the efficacy of anti-HER2 CAR-T cells against HER2-positive solid tumors (72). In a prostate cancer model, treatment with an oncolytic adenovirus armed with a helper-dependent adenovirus expressing a PD-L1 mini-antibody combined with HER2-CAR-T cells resulted in an enhanced anti-tumor effect both *in vitro* and *in vivo*. Additionally, delivering ICIs through oncolytic virus carriers proved superior to systemic administration of ICIs (73). In head and neck squamous cell carcinoma xenograft models, Rosewell et al. engineered a construct encoding the PD-L1-blocking antibody and IL-12p70, finding that adenovirus-derived IL-12p70 prevents the loss of HER2-CAR-T cells at the tumor site. This strategy augmented the anti-tumor effects of HER2-CAR-T cells, effectively controlling the growth of both primary and metastasized tumors (74). Researchers also analyzed an ongoing phase I clinical trial evaluating autologous HER2 CAR-T therapy for metastatic rhabdomyosarcoma in children. Although the patient initially showed a persistent response to HER2 CAR-T cells, cancer recurrence occurred six months after stopping HER2 CAR-T cell transfusion. After receiving HER2 CAR-T treatment again, the patient achieved a second complete remission and subsequently underwent additional CAR-T cell transfusion combined with the PD-1 antibody pembrolizumab. This child has remained healthy and cancer-free for 20 months since stopping HER2 CAR-T cell therapy (75).

The accumulation of abnormal metabolites in tumors, as well as typical conditions such as hypoxia, low pH, and oxidative stress, constitute the metabolic microenvironment that impairs the anti-tumor effect of CAR-T. For such abnormal metabolic microenvironments, modifying CAR-T or combining it with related inhibitors can effectively improve CAR-T efficacy. Adenosine is one of the highly accumulated metabolites in the tumor microenvironment, which inhibits CAR-T function by activating the surface adenosine receptor (A2aR) of CAR-T cells. Researchers have found that both simultaneous expression of anti-A2aRshRNA sequence in CAR-T structures and combination of CAR-T with A2aR specific small molecule antagonist SCH-58261 can effectively enhance CAR-T function (76). Researchers have also developed a CAR-T overexpressing catalase, which efficiently catalyzes hydrogen peroxide into water and oxygen while reducing the accumulation of reactive oxygen species, thereby maintaining a low oxidative state and exhibiting significant anti-tumor activity (77).

In addition, some solid tumors can inhibit the secretion of certain chemokines. The interaction between chemokines and their receptors promotes the migration of T cells into the tumor microenvironment. Meanwhile, the surface of CAR-T cells also lacks relevant receptors that match the chemokines secreted by solid tumors, resulting in poor homing ability of CAR-T to the tumor site. A study has modified the chemokine receptor CXCR5 onto the surface of CAR-T cells for the treatment of non-small cell lung cancer. The results of *in vivo* CAR-T tracking experiments showed that CAR-T cells modified with CXCR5 can migrate and infiltrate toward tumor lesions in a targeted manner, significantly clearing the tumor while greatly reducing potential extratumoral toxicity (78).

Currently, many researchers have overcome tumor microenvironment barriers through gene editing or metabolic reprogramming to improve the function of CAR-T cells. CRISPR/Cas9 gene editing technology is currently the most widely used gene editing technique for modifying CAR-T cells. And the latest researchers have explored the CRISPR-Cas13d editing system, a gene editing strategy that focuses on RNA rather than DNA. Through this strategy, researchers can achieve large-scale adjustments in gene expression without altering the core genetic information of cells, thereby successfully improving the function of CAR-T cells (79). In addition, researchers have found that overexpression of glucose transporter GLUT1 in CAR-T cells induces extensive metabolic reprogramming associated with increased glutathione mediated reactive oxygen species resistance and inosine accumulation, enhancing the function of CAR-T cells (80). Another study designed BCKDK engineered CAR-T cells for reprogramming branched chain amino acids metabolism in the tumor microenvironment based on genotype and phenotype modifications, and found significant prolongation of mouse survival, differentiation of central memory cells, and increased proportion of CAR-T cells in peripheral circulation (81).

### 3.3 Limited persistence of CAR-T cells

The clinical responsiveness of CAR-T cells is related to the *in vivo* expansion ability and long-term persistent activity of functional CAR-T cells (82). Most CAR-T cells cannot display long-term anti-tumor responsiveness as they almost inevitably experience fatigue or apoptosis after antigen induced activation (83, 84). Researchers have summarized strategies that can enhance the persistent responsiveness of CAR-T cells and improve their immune phenotype (85, 86).

Regarding the HER2 target, a promising strategy to enhance the persistence of adoptively transferred T cells involves expressing CARs in virus-specific T cells (VSTs) targeting cytomegalovirus, Epstein-Barr virus, or adenovirus, which are genetically modified to express HER2-CARs with a CD28.ζ-signaling endodomain. Clinical evidence indicates that HER2-CAR VSTs are both feasible and safe for treating progressive glioblastoma (87). A study has used CRISPR/Cas9 gene editing technology to knock out or inhibit IL-2-induced T cell kinases, and found that it can significantly reduce CAR-T cell failure, promote the formation of memory CAR-T cells, prolong the survival time of CAR-T cells *in vivo*, and ultimately improve tumor treatment efficacy (88). In CAR-T cell therapy, the percentage of T cell subsets with memory phenotype is positively correlated with better clinical response, while patients with poor response are more prone to depletion of T cells in their bodies and cannot exert lasting effects. Inhibition of FOXO1, the main regulatory factor of T cell memory imprints in CAR-T cells, will lead to cell exhaustion and reduce anti-tumor response, while increasing FOXO1 expression will enhance anti-tumor ability and increase mitochondrial mass (89). At the same time, co culturing cytokines such as IL-7 and IL-15 with CAR-T cells can promote CAR-T cell proliferation and differentiate CAR-T cells into memory T cells that are less glucose dependent and can exist for a long time, thereby enhancing the long-lasting killing efficacy of CAR-T cells (90, 91).

Numerous studies have shown that sustained metabolic disorders can lead to T cell exhaustion. Therefore, metabolic reprogramming is used to optimize cellular function and enhance anti-tumor efficacy. For example, enhancing glucose metabolism, regulating amino acid metabolism, resisting metabolic stress, thereby promoting the proliferation and function of CAR-T cells, and improving the adaptability and persistence of CAR-T cells in the tumor microenvironment (92). There are studies that modify T cells through engineering methods to enable CAR-T cells to secrete IL-10 on their own. When CAR-T cells enter a state of exhaustion, these new types of CAR-T cells can secrete and absorb IL-10 on their own, enhance mitochondrial oxidative phosphorylation metabolism, replenish energy, slow down and prevent T cell exhaustion, thereby increasing the killing power and persistence of CAR-T cells (93).

### 3.4 The safety of CAR-T cell therapy

In CAR-T cell therapy, patients may experience serious clinical complications. High levels of CAR-T cell expansion and target cell killing in a short period of time can lead to cytokine release syndrome (CRS).

A study has revealed the mechanism of CRS in CAR-T therapy, where CAR-T recognizes target cells and releases perforin and granzyme B, activating the caspase3-GSDME pathway and leading to cell pyroptosis and CRS. Pyroptotic cells can stimulate macrophages to activate caspase1-GSDMD and produce IL-6 and IL-1 β, thereby triggering CRS (94). Research has shown that catecholamine blockers can inhibit the release of inflammatory factors by macrophages (95). Therefore, it is speculated that simultaneously blocking catecholamines and GSDME may improve the safety of CAR-T therapy. Tocilizumab was approved by the FDA in August 2017 for the treatment of CRS following CAR-T immunotherapy, with significant efficacy. A team has designed a hypoxia inducible CAR-T (HiCAR) targeting the low oxygen characteristics of the tumor microenvironment. This CAR-T is driven by hypoxia response elements and includes an oxygen dependent degradation domain. It degrades under normoxic conditions and remains stable under hypoxic conditions. This feature not only enhances its toxicity to tumor cells under hypoxic conditions, but also ensures safety (96). Researchers have summarized some new safety switches based on small molecule CAR-T cells that are currently being studied, including FITC and folate, rimiducid, Rapamycin and other drugs are used to reduce the toxicity associated with CAR-T cell therapy (97). Some studies also use tetracycline based gene expression control systems to conditionally induce CAR gene expression (98, 99). Some researchers are also committed to achieving the “suicide” pathway of CAR-T by inducing suicide gene expression through the use of small molecule drugs (100). However, the effectiveness of these designs in improving safety while reducing toxicity still needs to be observed in clinical trials.

Although CAR-T faces many challenges in the treatment of solid tumors, with the continuous deepening of understanding of the characteristics of solid tumors and CAR-T cells, innovative solutions have emerged, bringing new hope to this therapy. Whether it is the optimization and modification of CAR-T’s own structure or the combination of CAR-T with other tumor treatment methods, both have shown good application prospects. CAR-T cell therapy is expected to achieve results in the field of solid tumors and hematological tumors, ultimately benefiting clinical cancer patients the most large scale.

## 4 HER2-CAR-T cell in human solid tumor therapy

*In vitro*, HER2-CAR-T cells exhibit robust targeted killing activity against a variety of solid tumors, including ovarian cancer (101), breast cancer (102), gastric cancer (33), pancreatic cancer (103), melanoma (104), glioblastoma (105), and head and neck cancer (106). Consequently, over the past decade, numerous clinical trials have been conducted to assess the safety and efficacy of HER2-CAR-T cells in treating these malignancies.

In a clinical trial using third-generation HER2-CAR-T cells to treat a patient with metastatic colon cancer exhibiting HER2 overexpression, the patient succumbed 5 days post-treatment. Serum samples taken after cell infusion revealed significant increases in multiple cytokines, including interferon-γ (IFN-γ), granulocyte-macrophage colony-stimulating factor (GM-CSF), tumor necrosis factor-α (TNF-α), IL-6, and IL-10, consistent with a cytokine storm (50). Therefore, optimizing the structure of HER2-CAR-T to significantly reduce the risk of cytokine storm while maintaining its killing effect is crucial. For example, using low affinity HER2 antibodies or reducing the 3 immunoreceptor tyrosine-based activation motifs (ITAMs) of CD3ζ in the CAR structure to 1. A phase I/II clinical study on second-generation HER2-CAR-T cell therapy in patients with recurrent/refractory HER2-positive sarcoma reported no significant toxic side effects when the infusion cell volume reached 1×10^8^/m^2^. Among the 19 infused patients, the median overall survival was 10.3 months, with four out of 17 evaluable patients exhibiting stable disease for 12 weeks to 14 months. HER2-CAR-T cells persisted for at least 6 weeks in 7 patients (107). Recently, the team conducted a phase I clinical study (ClinicalTrials.gov ID: NCT00902044) on HER2 positive advanced sarcoma patients found that the combination of fludarabine and cyclophosphamide with lymphocyte clearance significantly increased the expansion and duration of HER2 CAR-T cells. Although the CAR-T cell levels of most patients significantly decrease at week 6, repeated infusion of CAR-T cells can improve the bioavailability of CAR-T cells and have good tolerability. Among the 14 patients receiving treatment, 7 (50%) had stable or relieved disease, indicating that autologous HER2 CAR-T cell therapy for advanced sarcoma patients is safe and has certain anti-tumor activity after lymphocyte clearance (108). In the clinical trial of HER2-CAR VSTs for progressive glioblastoma, 8 out of 17 patients experienced clinical benefits, with 1 patient having a partial response lasting over 9 months, and 7 patients maintaining stable disease for 8 weeks to 29 months. The median overall survival for the entire cohort was 11.1 months (95% CI, 4.1-27.2 months) from the first T-cell infusion and 24.5 months (95% CI, 17.2-34.6 months) from diagnosis. This study developed a second-generation HER2-CAR based on the outer domain of FRP5 and the inner domain of CD28ζ to enhance safety, and relied on VST optimization for CAR-T cell persistence (87). In a phase I clinical trial of glioblastoma multiforme (GBM) (ClinicalTrials.gov ID: NCT01109095), researchers engineered HER2-CAR into T cells pre-selected for their ability to recognize cytomegalovirus (CMV). This initial evaluation of autologous HER2-CAR CMV bispecific T cells in patients with progressive GBM demonstrated safety, with cells persisting in the peripheral blood for up to 12 weeks. Clinical benefits were observed in 33% of patients, paving the way for combining HER2-CAR CMV T cells with other immunomodulatory approaches to enhance their expansion and anti-GBM activity. In a phase I clinical trial for HER2-positive advanced BTC and PC, 11 patients with advanced unresectable, recurrent, or metastatic cancer received CAR-T cell immunotherapy targeting HER2. Most patients experienced therapeutic effects, with mild-to-moderate adverse events related to the infusion of CAR-T-HER2 cells. The median progression-free survival was 4.8 months (range, 1.5–8.3 months). This study confirmed the safety and feasibility of CAR-T-HER2 immunotherapy, showing encouraging clinical activity signals (64). Clinical trials in central nervous system (CNS) tumors have shown that HER2-specific CAR-T therapy is tolerable in children with CNS tumors. However, no CAR-T cells were detected in the cerebrospinal fluid (CSF) or peripheral blood of the three participants, despite local inflammation following CNS injection (34). The above clinical trial results indicate that HER2 targeted specific CAR-T cell therapy has a broad application prospect in solid tumors, and has shown good therapeutic effects in various solid tumors. However, due to the heterogeneous expression of HER2 in different solid tumors, further exploration is needed to optimize HER2-CAR-T, whether in target development or CAR structure modification, in order to demonstrate better therapeutic effects. A summary of the above clinical trials is presented in **Table 1**.

**Table 1:** Summary of reported clinical trials on CAR-T cells targeting HER2-positive solid tumors.

Cancer	HER2-CAR structure	Study phase	Participant number	Results
Metastatic colon cancer	Third-generation	I	1	The patient succumbed to a cytokine storm five days following HER2-CAR-T cell therapy.
Sarcoma	Second-generation	II/III	19	HER2-CAR-T cells demonstrated persistence for 6 weeks without evident toxicities. The 19 patients who received infusions exhibited good tolerance, with a median overall survival of 10.3 months (ranging from 5.1 to 29.1 months).
Sarcoma	Second-generation	I	14	50% of patients have stable or improved disease. The 1-year survival rate is 46%, and the median follow-up time is 8.2 months. One patient achieved complete remission after receiving multiple CAR-T cell infusions and maintained remission for over 6 years.
Glioblastoma	Second-generation	I	17	Seventeen patients exhibited good tolerability with no dose-limiting toxic effects; eight of these patients achieved clinical benefits. The median overall survival from the first infusion of T cells was 11.1 months (95% CI, 4.1-27.2 months).
Glioblastoma multiforme (GBM)	Second-generation	I	16	Autologous HER2-CAR cytomegalovirus (CMV) bispecific T-cell therapy for patients with progressive GBM has demonstrated safety, with cells persisting in peripheral blood for 12 weeks and clinical benefits observed in 33% of patients.
BTC and PC	Second-generation	I	11	Adverse events related to the infusion of CAR-T HER2 cells were mild to moderate. One patient achieved a partial response lasting 4.5 months, and five patients achieved stable disease. CAR-T HER2 immunotherapy was deemed safe and feasible.
CNS	Second-generation	I	3	The patients experienced no dose-limiting toxicity and exhibited clinical, as well as correlative laboratory, evidence of local CNS immune activation, including high concentrations of CXCL10 and CCL2 in the cerebrospinal fluid.

As shown in **Table 2**, numerous clinical trials for HER2-CAR-T cell therapy targeting solid tumors have been registered on clinicalins.gov (109). One clinical trial (NCT04650451) assessed the safety, tolerability, and clinical activity of HER2-specific dual-switch CAR-T cells, BPX-603, administered with rimiducid to participants with previously treated, locally advanced, or metastatic HER2-amplified/overexpressed solid tumors. Recruitment was suspended on April 20, 2023, due to dose-limiting toxicity observed in a related trial using the same technology. The current strategy to solve this problem is mainly to modify the CAR structure, not excessively activate the release of cytokines, and minimize the risk of cytokine storms. Alternatively, safety switches can be added to the CAR structure to effectively block cytokine storms, thereby improving the safety of CAR-T clinical treatment. Currently, 12 clinical studies involving HER2-CAR-T cells are actively recruiting patients; 5 are conducted in China and 7 in the United States. Clinical trial NCT04511871 involves CCT303-406 (a HER2 antagonist) CAR-modified autologous T cells, evaluating their safety, tolerability, and anti-tumor activity in patients with recurrent or refractory HER2-positive solid tumors. The safety and efficacy of anti-HER2-CAR-T cell injection therapy for HER2-positive locally advanced and/or metastatic solid tumors are evaluated in phase I clinical trial NCT06101082. Furthermore, a novel hypoxia-stimulated CAR expression system (HypoSti.CAR) has been developed to enable CAR-T cells to effectively expand and survive in hypoxic tumor microenvironments. The safety, feasibility, and efficacy of HypoSti.CAR-HER2 T cells in HER2 antigen-positive advanced solid tumors are assessed in clinical trial NCT05681650. A research team has constructed third-generation CAR-T cells targeting various antigens such as GPC3, mesothelin, claudin18.2, GUCY2C, B7-H3, PSCA, PSMA, MUC1, TGFβ, HER2, Lewis-Y, AXL, and EGFR. They are testing the safety, tolerability, and preliminary efficacy of these individual or combination CAR-T cells in phase I clinical study NCT03198052. Additionally, phase I clinical trial NCT04684459 investigates HER2/PD-L1 dual-targeting CAR-T cells in patients with HER2-positive solid tumor serosal cavity metastases. In this study, PD-L1 targeted chimeric switch receptor (PD-L1. BB CSR) can bind to PD-L1 antigen in the environment of malignant pleural effusion or ascites, converting inhibitory signals into 4-1BB signals. By simultaneously expressing PDL1.BB CS modified dual targeted CAR-T cells on T cells with second-generation tumor associated antigen-specific CARs, PDL1.BB CS modified dual targeted CAR-T cells exhibit enhanced adaptability and function in patient derived malignant pleural effusion or ascites. More importantly, the regional delivery of dual targeted CAR-T cells can enable rapid and persistent eradication of pleural and peritoneal metastases in xenograft models (32).

**Table 2:** Clinical trials of HER2-CAR-T cell therapy in solid tumors (ClinicalTrials.gov).

Cancer	Clinical trial identifier	Study phase	Expected participant number	Biological	Status
HER2-positive gastric cancer, breast cancer, and HER2 protein overexpression solid tumor	NCT04650451	I/II	220	BPX-603	Suspended
Gastric cancer, breast cancer, ovarian cancer, sarcoma	NCT04511871	I	15	CCT303–406	Recruiting
Solid tumor	NCT06101082	I	9	HER2-CAR-T cells	Recruiting
HER2 positive advanced solid tumors	NCT05681650	I/II	30	HypoSti.CAR-HER2 T cells	Recruiting
Lung cancer	NCT03198052	I	30	CAR-T cells targeting GPC3, Mesothelin, Claudin18.2, GUCY2C, B7-H3, PSCA, PSMA, MUC1, TGFβ, HER2, Lewis-Y, AXL, or EGFR	Recruiting
HER2 positive solid tumor serosal cavity metastases	NCT04684459	I	18	HER-2/PD-L1 dual targeted CAR-T cells	Recruiting
Solid tumor	NCT03740256	I	45	Combination of CAdVEC and HER2- CAR-T cells	Recruiting
Advanced sarcoma	NCT00902044	I	36	HER2-CAR-T cells	Recruiting
Metastatic malignant neoplasm in the brain or leptomeninges, HER2-positive breast cancer	NCT03696030	I	39	HER2-CAR-T cells	Recruiting
Advanced solid tumor	NCT06241456	I	351	FT825/ONO-8250	Recruiting
Ependymoma	NCT04903080	I	50	HER2-CAR-T cells	Recruiting
Advanced sarcoma	NCT04995003	I	25	Combination of HER2-CAR-T cells and pembrolizumab or nivolumab	Recruiting
Pediatric diffuse intrinsic pontine glioma, diffuse midline glioma, recurrent or refractory central nervous system tumors	NCT05768880	I	72	SC-CAR4BRAIN	Recruiting
Metastatic breast cancer	NCT06251544	I	27	TRAIL-R2 and HER2 bi-specific CAR T cells	Not yet recruiting
Solid tumor	NCT05745454	I	12	HER2-E-CAR-T cells	Not yet recruiting
Recurrent/refractory brain tumor	NCT02442297	I	10	HER2-CAR T cells	Active, not recruiting
Recurrent/refractory malignant glioma	NCT03389230	I	29	HER2(EQ)BBζ/CD19t+ T cells	Active, not recruiting
HER2 positive recurrent/refractory pediatric central nervous system tumors	NCT03500991	I	10	HER2-CAR T cells	Active, not recruiting
Pancreatic Cancer	NCT03267173	I	10	CAR T cells	Unknown
Breast cancer	NCT04430595	I/II	100	4SCAR T cells	Unknown
HER2 positive malignancies	NCT00889954	I	20	TGF-β resistant HER2/EBV-CTLs	Completed

In the clinical trial NCT03740256, the efficacy of a binary oncolytic/helper-dependent adenovirus (CAdVEC) combined with HER2-CAR-T cells was evaluated in patients with advanced HER2-positive solid tumors. CAdVEC can both lyse tumor cells and locally express pro-inflammatory cytokines IL-12 and PD-L1 blocking antibodies, while repolarizing the tumor microenvironment and increasing T cell infiltration (110). Additionally, researchers sought to determine the maximum safe dose of HER2-CAR-T cells through clinical trial NCT00902044, assessing their potential benefit for patients with sarcoma and the safety of HER2-CAR-T cell therapy following lymphatic depletion chemotherapy. Another study (NCT03696030) investigated the treatment of patients with HER2-positive cancer exhibiting brain and/or leptomeningeal metastases *via* intraventricular administration of autologous HER2-CAR-T cells. A novel cancer-specific anti-HER2 antibody, H2Mab-250/H2CasMab-2 (IgG1, kappa), which exhibits high reactivity to HER2-positive breast cancer tissues without affecting normal tissues, has been developed (111). Martin Hosking’s research group described FT825/ONO-8250, an off-the-shelf CAR-T cell therapy engineered with the H2CasMab-2 binder and seven functional elements to overcome barriers in solid tumor cell therapy (112). Their phase I clinical trial (NCT06241456) aimed to evaluate the safety, tolerability, and antitumor activity of FT825/ONO-8250, with or without monoclonal antibody therapy, following chemotherapy in participants with advanced HER2-positive or other advanced solid tumors. FT825/ONO-8250 designed and developed based on Fate’s proprietary iPSC platform can be mass-produced, saving time, effort, and costs, meeting the needs of a wide range of patients, and thus can be widely used in clinical practice. A phase I study (NCT04903080) assessed the safety of intravenous HER2-specific CAR-T cell injections after lymphatic depletion chemotherapy in pediatric patients with refractory or recurrent ependymoma. Furthermore, clinical trial NCT04995003 aimed to determine the safety and side effects of administering HER2-CAR-T cells in combination with immune checkpoint inhibitors (pembrolizumab or nivolumab) and their potential efficacy in patients with sarcoma. Researchers also developed second-generation CAR-T cells targeting B7H3, EGFR806, HER2, and IL13 zetakine, termed SC-CAR4BRAIN, and subsequently conducted a phase I study (NCT05768880) to evaluate SC-CAR4BRAIN for local adoption therapy in the CNS.

Five phase I clinical trials (NCT06251544, NCT05745454, NCT02442297, NCT03389230, NCT03500991) are currently in the initiation phase but have not yet begun recruitment. Investigators utilized a retrovirus to insert HER CAR, TR2 antibody, and IL-15 genes into T cells, aiming to determine the maximum safe dose of TRAIL-R2 and HER2 bi-specific CAR T cells, monitor their persistence in the body, identify side effects, and evaluate their efficacy against HER2-expressing cancers. The safety and tolerability of HER2-E-CAR-T cells for treating HER2-positive, refractory advanced solid tumors are assessed across three dose groups: low, medium, and high. Tangdu Hospital conducted another clinical trial, optimizing the CAR structure based on traditional second-generation CAR-T cells and designing HER2-E-CAR-T cells. Effectiveness, *in vivo* persistence, and significant reduction of cytokine storms were verified through both *in vitro* and *in vivo* experiments. Another clinical trial seeks to determine the maximum safe dose of HER2-CAR T cells, understand their side effects, and evaluate their potential benefit for patients with brain tumors volunteering for this new treatment. A phase I study assessed the feasibility and safety of cellular immunotherapy using *ex vivo* expanded autologous memory-enriched T cells, genetically modified with a self-inactivating (SIN) lentiviral vector to express a HER2-specific, hinge-optimized, 41BB-costimulatory CAR, as well as a truncated human CD19 (HER2[EQ]BBzeta/CD19t+), for participants with recurrent/refractory malignant glioma. Another phase I study evaluated the efficacy of autologous HER2-CAR-T cells in treating refractory or recurrent central nervous system tumors, examining the distribution of CAR-T cells in CSF, their migration into peripheral circulation, and the degree of HER2 expression at diagnosis versus recurrence.

Additionally, two clinical trials (NCT03267173, NCT04430595) related to HER2-CAR-T cells have unknown status, and one (NCT00889954) has been completed. From June 2017 to June 2019, researchers evaluated the safety and efficacy of CAR-T cell immunotherapy targeting Mesothelin, PSCA, CEA, HER2, MUC1, and EGFRvIII for pancreatic cancer. Another phase I/II study assessed the feasibility, safety, and efficacy of multiple fourth-generation CAR-T cells targeting HER2, GD2, and CD44v6 surface antigens in breast cancer. The completed clinical trial treated patients with TGF-β resistant HER2/EBV-CTLs (EBV-specific cytotoxic T lymphocytes transduced to express the mutant type II TGF-β dominant-negative receptor and the HER2 CAR.

## 5 Conclusion and future direction

The HER2 receptor, recognized as a biomarker and a valuable therapeutic target, has led to the registration of over 2000 clinical trials evaluating novel HER2-targeted therapies. Recently, HER2-specific CAR-T cell therapy has gained prominence in HER2-positive solid tumor immunotherapy. Preclinical studies have demonstrated the potent anti-tumor effects of HER2-CAR-T cells, which have shown notable safety and moderate efficacy in clinical trials (113). However, these clinical benefits are often accompanied by significant immune-mediated toxicities, such as cytokine release syndrome and immune effector cell-associated neurotoxicity syndrome (114). Moreover, several challenges hinder the successful application of CAR-T cells in solid tumors, including limited persistence, low therapeutic tolerability, inefficient trafficking to tumor sites, a hostile tumor microenvironment, and the risk of on-target/off-tumor toxicities (14). To address these issues, researchers are adopting various strategies to enhance HER2-CAR-T cell functionality (**Figure 3**). Innovations include the development of CAR macrophages (HER2-CAR-M) (115–117) and natural killer cells (HER2-NK) (118) targeting HER2 for solid tumor treatment. As scientific research and technology advance, HER2 remains a versatile target, offering increasing benefits through differentiated and combination therapies. These therapies encompass conventional monoclonal antibodies, as well as novel agents such as ADCs, bispecific antibodies, CAR-T, and CAR-M cell therapies. In addition, there are currently many CAR-T clinical targets under development for solid tumors, including Claudin 18.2, MSLN, GPC3, EGFR, GD2, CD133, PSCA, and other popular solid tumor targets. Combining HER2 with such specific targets may become a strategy to improve CAR-T efficacy. For example, syNotch technology can be used to combine HER2 with another target, and only when both antigens are expressed simultaneously in tumor cells, CAR-T will be activated to exert its killing function. However, there are currently few CAR-T studies combining HER2 with these targets, which may be related to the overlap rate of dual antigen expression in solid tumors. For example, in gastric cancer patients with high expression of CLDN18.2, only 12% express HER2, indicating that only 12% of gastric cancer patients may have a good response to HER2 and CLDN18.2 dual target CAR-T therapy.

At present, all CAR-T therapies approved for market worldwide are autologous products. Due to research and development costs and complex preparation processes, the production cost of autologous CAR-T therapies remains high. A study reported for the first time the use of a allogeneic universal CAR-T developed by Bangyao Biotechnology to treat rheumatic immune diseases, helping three patients with rheumatic immune diseases achieve long-term remission (119). This study suggests that there is potential to expand the production scale of universal CAR-T therapy cells, which may significantly reduce costs and production time in the future and meet the needs of large-scale patients. Universal CAR-T cells can be produced in advance, making it easier to undergo *in vitro* procedures and improving treatment efficiency. For patients who cannot obtain sufficient quantity or quality of autologous CAR-T cell products, universal CAR-T cells provide an alternative solution. Universal CAR-T can reduce the occurrence of graft versus host disease (GvHD) and improve the persistence of CAR-T cells in patients by removing TCR and MHC-I molecules from CAR-T cells through gene editing technology. Universal CAR-T endows CAR-T cells with immune escape ability. These advantages make universal CAR-T cells an important direction for future cancer treatment (120). However, the safety of allogeneic CAR-T still needs to be evaluated, especially regarding the potential for GvHD. To solve the problem of immune rejection, knocking out the TCR of allogeneic CAR-T through gene editing technology is the most crucial step. To avoid rejection reactions of transduced pre-existing T cells, gene editing techniques can also be used to knock out MHC class I molecules from the donor source, or to clear T cells and NK cells from the patient’s body through CD52 monoclonal antibodies or CD7 CAR. In addition, the development of allogeneic CAR-T using non-gene editing techniques can avoid the off target risk of gene editing, and the expression of CAR and the elimination of GvHD can be achieved simultaneously using a single viral vector. Allogeneic CAR-T also poses safety issues such as CRS, neurotoxicity, and infection. However, compared with the currently released clinical trial data, it can be found that allogeneic CAR-T has good safety, with an incidence of adverse reactions close to or even lower than autologous CAR-T. The currently announced CD19 allogeneic CAR-T response rates are good, and some products such as CB-010 and PBCAR0191 can even achieve an objective response rate of 100% (121). In addition, researchers have developed a transient engineered CAR-T cell therapy generated *in vivo*. By injecting mRNA delivered by lipid nanoparticles (LNP), T cells are reprogrammed to recognize myocardial fibrotic cells, thereby reducing fibrosis and restoring heart function in a mouse model of heart failure (122). This method may have the potential to solve the current challenges of complex CAR-T therapy processes, long cycles, and high prices. However, more time is needed to demonstrate that the newly developed CAR-T therapy is as effective and safe as, or even superior to, existing autologous CAR-T cell therapies.

In addition, the development and commercialization of CAR-T cell therapy are accompanied by complex regulatory requirements and challenges (123). In fact, since the development and approval of CAR-T therapy, some common adverse reactions have been addressed and properly handled. Until last year, following the FDA’s attention to the potential carcinogenic risks of autologous CAR-T therapy targeting BCMA or CD19, regulatory agencies in multiple countries and regions around the world increased their focus on CAR-T therapy. At present, the measures taken by regulatory agencies in various countries may affect the development progress of CAR-T therapy in two aspects. One is that although clinical trials will proceed as planned, they may take longer to complete, and the other is that CAR-T therapy may face stricter scrutiny during the marketing process. CAR-T therapy represents a new era in cancer treatment, and its significant efficacy in certain types of cancer treatment cannot be ignored. With the in-depth research and application of this treatment method, its potential risks also need to be carefully addressed.

In China, although CAR-T therapy started late, its development speed is extremely fast. With more CAR-T products approved, the market size is expected to reach 28.9 billion yuan by 2030. However, since the launch of China’s first CAR-T, the high cost of treatment has deterred many patients until now. In recent years, many companies in China have been constantly trying to reduce the cost of CAR-T therapy and increase product penetration through various means. For example, cost control during the research and development phase, optimization of supply chain management and reduction of losses, cost reduction and improvement of production efficiency during the production process, as well as strengthening cooperation with the government and enterprises. The balance between the price and value of innovative drug CAR-T has not yet been found to be the optimal solution, but there is no doubt that an innovative drug that only meets scientific value is far from enough. When it transforms from a molecule into a drug, both patient value and social value must be considered. We hope to have a safe, effective, and affordable immune cell therapy product in the future that can provide treatment and survival benefits to a large number of patients.
